# Trade-offs between robustness and small-world effect in complex networks

**DOI:** 10.1038/srep37317

**Published:** 2016-11-17

**Authors:** Guan-Sheng Peng, Suo-Yi Tan, Jun Wu, Petter Holme

**Affiliations:** 1 College of Information System and Management, National University of Defense Technology, Changsha, Hunan 410073, P. R. China; 2 Department of Computer Science, University of California, Davis, California 95616, USA; 3 Department of Energy Science, Sungkyunkwan University, 440-746 Suwon, Korea

## Abstract

Robustness and small-world effect are two crucial structural features of complex networks and have attracted increasing attention. However, little is known about the relation between them. Here we demonstrate that, there is a conflicting relation between robustness and small-world effect for a given degree sequence. We suggest that the robustness-oriented optimization will weaken the small-world effect and vice versa. Then, we propose a multi-objective trade-off optimization model and develop a heuristic algorithm to obtain the optimal trade-off topology for robustness and small-world effect. We show that the optimal network topology exhibits a pronounced core-periphery structure and investigate the structural properties of the optimized networks in detail.

Many real-world complex systems can be modeled as networks. Examples include the Internet, metabolic networks, electric power grids, supply chains, urban road networks, and the world trade web among many others. In the last decades, an emergent science of networks has attempted to explain, predict, and control these networked systems. The study of complex networks has become an important area of multidisciplinary research involving physics, mathematics, biology, social sciences, informatics, and other theoretical and applied sciences^1^,^2^,^3^,^4^,^5^,^6^.

The function and behavior of networked systems can be largely influenced by their structural features. As one of the fundamental structural features, network robustness (also known as resilience, tolerance, survivability, invulnerability), i.e., the ability of a network to maintain its connectivity when a fraction of nodes (links) is damaged, has received growing attention in many fields^7^,^8^,^9^. In ecology, network robustness is an important attribute of ecosystems, and can give insight into the reaction to disturbances such as the extinction of species^10^. For biologists, network robustness can help the study of diseases and mutations, and how to recover from some mutations^11^. In economics, network robustness principles can help our understanding of the stability and risks of banking systems^12^. And in engineering, network robustness can help to evaluate the resilience of infrastructure networks such as the Internet or power grids^13^. Many studies have been devoted to designing robust networks^14^,^15^,^16^,^17^,^18^,^19^,^20^,^21^ motivated by applications in communication networks^22^, power grid^23^, transportation network^24^, sensor networks^25^, logistic network^26^ and so on. However, many real networks are the results of complex and extended processes; thus, designing networks from scratch is practically impossible. Therefore, the study of improving existing networks is of great interest. Recently, some methods for enhancing network robustness by modifying the topology have been proposed, e.g., adding links^27^,^28^, deleting^29^, or rewiring links^30^,^31^,^32^,^33^,^34^. However, despite recent research focusing on the enhancement of network robustness, little has been done on the joint optimization of network robustness and other desirable structural features. This is the motivation for our study.

In this paper, along with the network robustness, we focus on another important and well-known structural feature: small-world effect, the fact that most pairs of nodes are connected by a relatively short path through the network^2^. The existence of the small-world effect had been speculated upon before the Milgram’s experiment and the more rigorously in the mathematical work of Pool and Kochen^35^. Nowadays, the small-world effect has been studied and verified directly in a large number of different networks. The small-world effect has obvious implications for the diffusion processes taking place on networks. For example, if one considers the spread of information across a network, the small-world effect implies that the spread will be fast on most real-world networks. If it takes only six steps for a rumor to spread from any person to any other, for instance, then the rumor will spread much faster than if it takes a hundred steps, or a million. Moreover, small-effect effect is also related to the synchronization of oscillator networks^36^. The small-world effect has been used across the many diverse applications^37^. The small-world effect is useful in analysis of man-made networks such as transportation networks and communications networks. It is used to help determine how cost-efficient a particular network construction is. Beyond human constructed networks, the small-world effect is a useful metric when talking about physical biological networks. For example, it is used in neuroscience to discuss information transfer across neural networks, where the physical space and resource constraints are a major factor^38^. There are some previous works addressing the relation between robustness and small-world effect. For example, Netotea and Pongor studied the evolution of robust yet small-world network topologies and how the selection for robustness or small-world effect influences network topology^39^. Brede *et al.* studied networks that optimize a trade-off between small-world effect and resilience^40^. However, these works did not preserve node degrees. For the practical purposes, changing the degree of a node can be more expensive than changing the connection in networked system. For example, the adjustment of airline is more easier than increasing the capacity of airports. Therefore, conserving the degrees is a reasonable constraint that we will use.

Here we investigate the relation between robustness and small-world effect while keeping the degrees of each node fixed in optimization. We focus on the complex networks with non-trivial topological features that do not occur in simple networks such as lattices or random graphs. To realize the trade-off between robustness and small-world effect, we build a multi-objective optimization model and develop a heuristic algorithm. We report on successful results from applying the method to scale-free network for discussing how robustness and small-world effect influence on network topology.

## Results

### Conflicting relation between robustness and small-world effect

**Figure 1:**
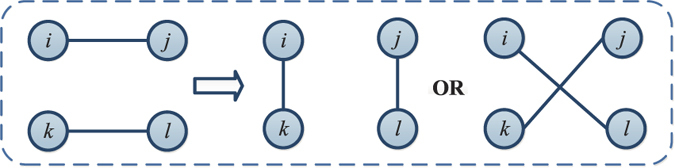
Degree-preserving rewiring process.

**Figure 2:**
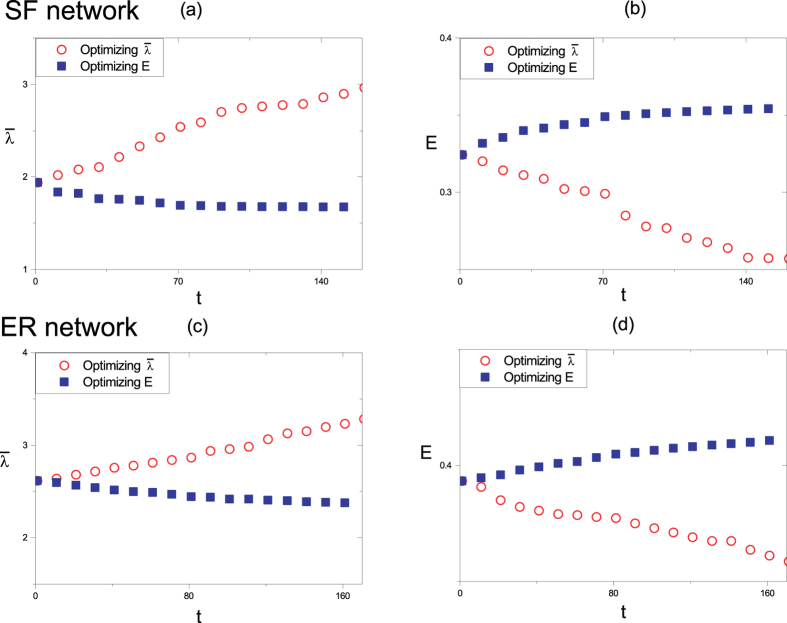
The change of the robustness 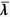 and small-world effect *E* versus iterations *t* in single-objective optimization in different model networks. (**a**) and (**b**) correspond to scale-free network with its power-law exponent *γ* = 3, while (**c**) and (**d**) correspond to ER random network with its connection probability *p* = 0.05. The network size is *N* = 100.

In this paper, we use a spectral measure of robustness—natural connectivity 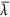. It measures the robustness of a network by quantifying the redundancy of paths between each pairs of nodes, and has been proved to sensitively exhibit the variation of the robustness^41^,^42^. Besides, a measure *E* defined as the reciprocal harmonic average of shortest distances is adapted to describe the extent of small-world effect^43^. To analyze the relation between robustness and small-world effect, we first propose a single-objective optimization model to optimize robustness and small-world effect separately and then investigate the change of one property when optimizing another one. We consider scenarios where changing the degree of a node is significantly more expensive than rewiring a link, thus we impose the constraint that each node degree *d*_*i*_ is fixed in optimization process. Besides, the optimized network should be connected. We use the degree-preserving greedy optimization algorithm^44^,^45^ shown in Fig. 1 to search the optimal robust network or the optimal network with small-world effect. Starting from an initial network, we adopt the rewiring method at each time step. Only if the objective is improved and the generated network is still a connected simple network, the rewiring is accepted. We repeat this procedure for many iterations to obtain the optimized network. We apply the single-objective optimization method to model networks. Figure 2 shows the change of the robustness 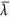 and the small-world effect *E* versus iterations *t* in scale-free networks and ER random graphs, respectively. We find that optimizing 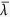 will reduce *E* obviously while increasing *E* also leads to a great loss of robustness 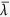. It means that robustness and small-world effect are in a conflicting relation.

**Figure 3:**
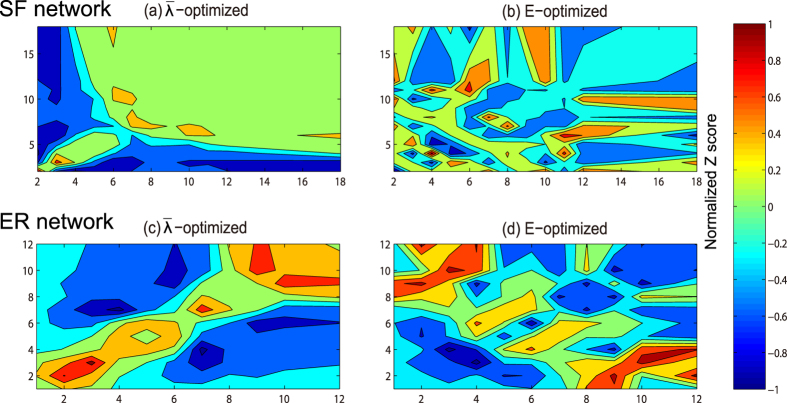
The correlation profiles of the 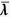-optimized network and the *E*-optimized network based on scale-free network and ER random network. The *Z* score value is normalized to a range of −1 to 1 and indicated by the color. The higher the value of *Z(d*_*i*_, *d*_*j*_) score is, the more links between *d*_*i*_-degree nodes and *d*_*j*_-degree nodes the network has.

Since the degree of each node is fixed in optimization, the degree correlation is a significant network property that deserve investigation. In a work by Milo *et al.*^46^, they presented an approach for comparing network local structure based on the significance profile (SP). To obtain the SP of a network, the statistical significance for each subgraph is calculated by comparing the network to an ensemble of randomized network with the same degree sequence. Similarly, this method can also be used to analyze the degree correlations in networks. Specifically, when studying the degree correlations, the statistical significance is described by the *Z* score:


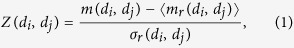


where *m(d*_*i*_, *d*_*j*_) is the number of links between the nodes with degree *d*_*i*_ and nodes with degree *d*_*j*_ in the network, and 〈*m*_*r*_(*d*_*i*_, *d*_*j*_)〉 and *σ*_*r*_(*d*_*i*_, *d*_*j*_) are the mean and standard deviation of the values of *m(d*_*i*_, *d*_*j*_) in a randomized network sets, where the networks are randomized from the specific network by executing the degree-preserving rewiring algorithm^44^ enough times. Therefore, according to the equation, *Z* score reflects the density of connections between nodes with different degrees. The higher the value of *Z(d*_*i*_, *d*_*j*_) score is, the more connections between *d*_*i*_-degree nodes and *d*_*j*_-degree nodes the network has. Figure 3 shows the correlation profiles of the 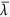-optimized network and the *E*-optimized network based on scale-free network and ER random network. The *Z* score value is normalized to a range of [−1, 1] and indicated by the color in the correlation profiles. The randomized network is generated by rewiring the original networks in Fig. 3 for 10^4^ times while keeping node degrees fixed. We generate 10^3^ randomized networks to ensure the stability of results. The results in Fig. 3(a) and (c) show that the optimization for 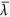 results in a more assortative topology compared to the randomized network with the same degree sequence. Nodes in 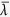-optimized network prefer to attach to those nodes with similar degree. On the contrary, the *E*-optimized network is inclined to be a disassortative network, in which high-degree nodes prefer to connect low-degree nodes rather than connect the nodes with similar degree. Therefore, the *E*-optimized network shows a high degree of difference with 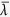-optimized network, which implies that robustness and small-world effect are in a conflicting relation.

**Figure 4:**
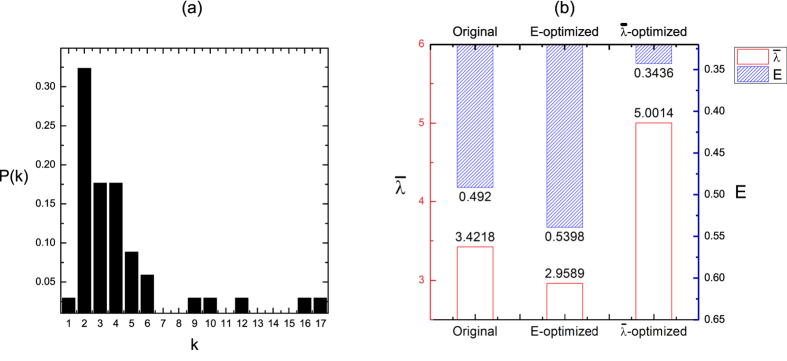
Optimization results on a real network. (**a**) The degree distribution of the real network. (**b**) The values of 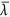 (red) and *E* (blue) before and after the optimization. The real network is the social network in a karate club at a US university in the 1970s. It has 34 nodes and 78 links. The length of column with red line corresponds to the value of 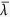 axis, while the column with blue shading corresponds to the *E* axis. The results are averaged over 100 independent realizations.

**Figure 5:**
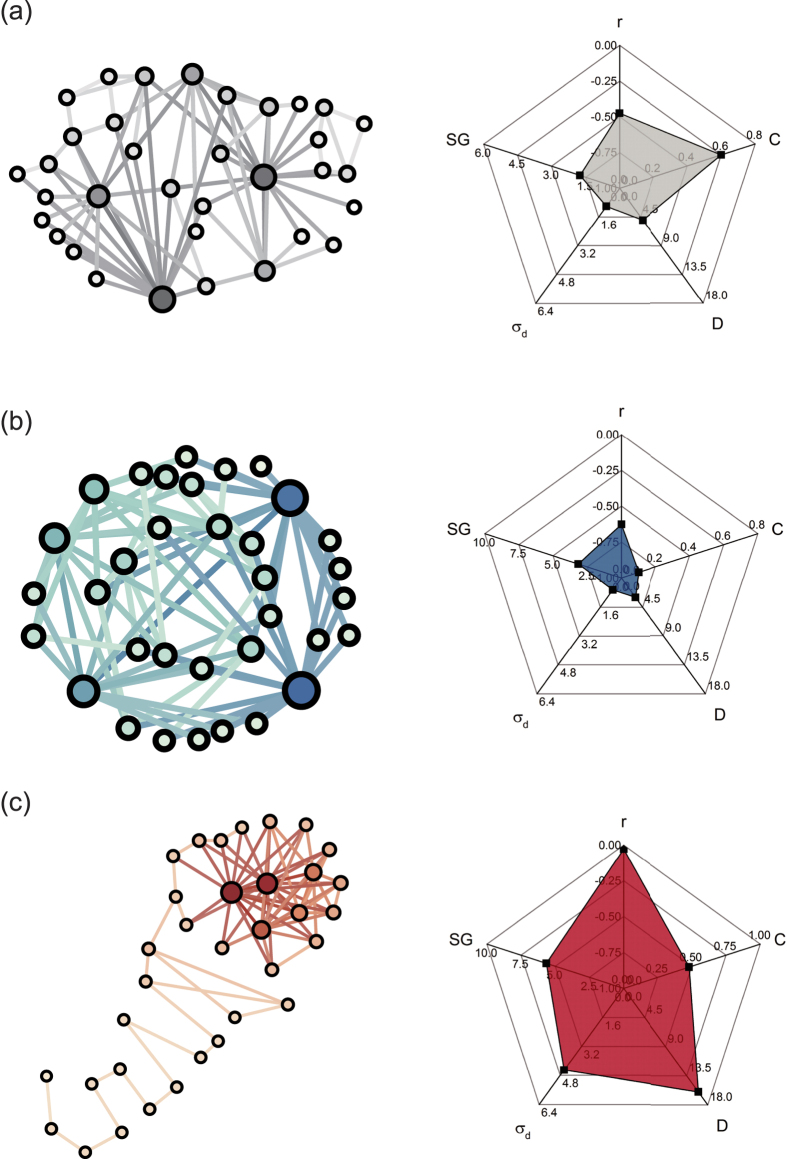
The visualization of original network and the optimized networks and their topological properties. (**a**) original network, (**b**) *E*-optimized and (**c**) 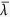-optimized network have the same degree sequence. The degree of node is proportional to its size. The radar graphs show the values of topology metrics including assortativity coefficient *r*, clustering coefficient *C*, network diameter *D*, standard deviation of distance distribution *σ*_*d*_ and spectral gap *SG*.

To verify the inferences, we also investigated the trial on a real network, Zachary’s karate club, which is a social network in a karate club at a US university in the 1970s^47^. It has 34 nodes and 78 links (see Fig. 4(a)). Figure 4(b) shows the value of 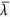 and *E* in the original network, the 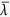-optimized network and *E*-optimized network. The results shows similar conclusions, i.e., optimizing 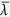 will result in a decrease of *E*, while maximizing *E* will decrease 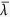 of network. Several primary topological properties are expressed through radar charts in Fig. 5. We can clearly see the different topologies of 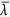-optimized and *E*-optimized networks. The *E*-optimized network (Fig. 5(b)) exhibits roughly a multi-hub, local star-like structure in which high-degree nodes (hubs) are interconnected through low-degree nodes, and low-degree nodes rarely attach to each other. In the multi-hub star-like structure, information on any node can be quickly transmitted to other nodes through the hubs they connected. Moreover, lower *D*, lower *σ*_*d*_ shown in radar chart illustrates that each pair of nodes have approximately the same short length of distance. The multi-hubs star-like network has a disassortative structure and a lower cluster coefficient. On the contrary, the 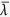-optimized network (Fig. 5(c)) has a core-chain with a core comprised of high-degree nodes and other low-degree nodes that are interconnected to form a long chain. The highly interconnected hubs guarantee the connectivity of network after the removals of some nodes, even the high-degree nodes, whereas the structure with a long chain composed of low-degree nodes will leads to a high cost of communication between nodes. The core-chain topology displays a high degree of assortativity and clustering. That multi-hub star-like represent the extreme case of disassortativity and the core-chain topology represent networks optimized for assortativity was found in ref. 48. The results in radar charts show that the robust networks and the efficient networks are completely different in our network metrics.

### Trade-off between robustness and small-world effect

**Figure 6:**
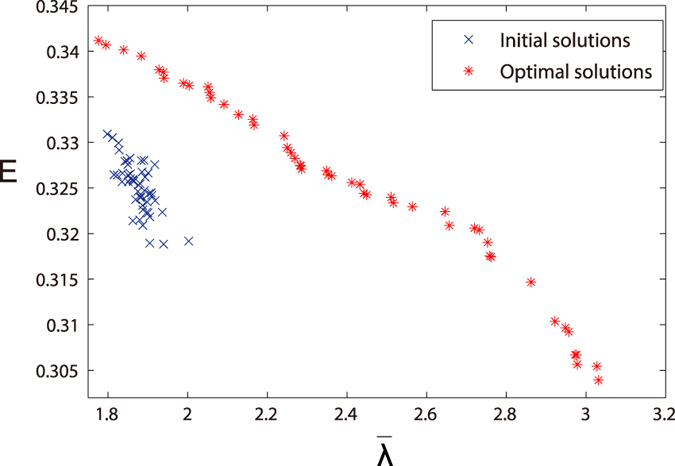
Pareto-optimal fronts in the multi-objective optimization for 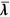 and *E*. The red points represent the optimized networks, and the blue points represent the original networks. The size of population is 50. Each solution in population is a scale-free network with 100 nodes, 179 links and power-law exponent *γ* = 3.

To consider both the robustness and small-world effect of network in optimization, SMS-MOEA is utilized to obtain the Pareto-optimal front, namely the best possible set of non-dominating solutions. In Fig. 6, we show the Pareto-optimal front of 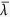 and *E* optimized from the initial solutions, which is generated from an initial scale-free network with nodes *N* = 100, links *M* = 179 and power-law exponent *γ* = 3 by executing the mutation operator for 10^3^ times. Here we state the parameter used for SMS-EMOA: The population size is 50, the crossover probability is *P*_*c*_ = 0.9, and the mutation probability is *P*_*m*_ = 0.05. Besides, we iterate the optimization 20,000 times. As expected, the index *E* of networks in Pareto-optimal front decreases monotonically with their robustness 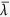 in Fig. 6. Hence, the results are well-distributed and have a good convergence, which vertify the effectiveness of SMS-MOEA when optimizing the network topology.

**Figure 7:**
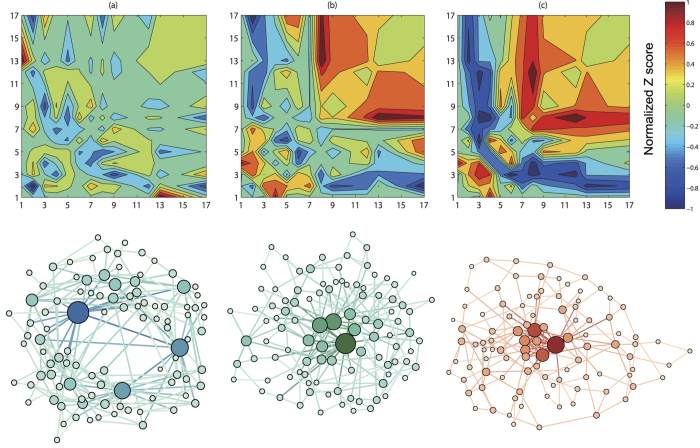
The correlation profiles and the visualization of selected networks in Pareto-optimal solutions set. (**a**) The network with high *E* and low 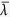. (**b**) The network with both relatively high 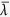 and *E*. (**c**) The network with high 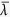 and low *E*. The *Z* score value is normalized to a range of −1 to 1 and indicated by the color. The higher the value of *Z(d*_*i*_, *d*_*j*_) score is, the more links between *d*_*i*_-degree nodes and *d*_*j*_-degree nodes the network has.

**Figure 8:**
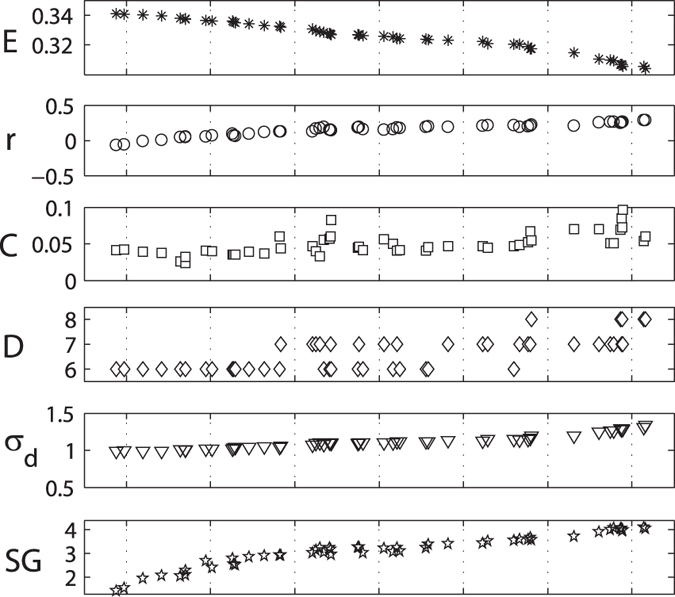
Topological properties of the optimized networks in Pareto-optimal solutions set. The assortativity coefficient *r*, clustering coefficient *C*, network diameter *D*, standard deviation of distance distribution *σ*_*d*_ and spectral gap *SG* are listed to understand their relation to robustness and small-world effect. These topological properties of networks are in ascending order of their value of 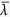.

We analyze the change in network structure as the optimization orientation for robustness and small-world effect are shifted. Figure 7 gives the correlation profiles and the exhibition of three selected networks in Pareto-optimal solutions. Figure 7(a) and (c) show the topology of the network with high *E* and the network with high 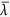 respectively, while the intermediate network with both high 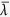 and *E* is shown in Fig. 7(b). In Fig. 8, the topological properties of optimized networks in Pareto-optimal front is depicted. Here we can clearly observe the transition from small-world network structure towards robust network structure.

As described above, the network with low 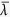 and high *E* roughly display a multi-hubs star-like structure, in which most of low-degree nodes are directly connected to high-degree nodes. Therefore, the network has low assortativity coefficient *r*, low clustering coefficient *C*, low network diameter *D* and low standard deviation of distance distribution *σ*_*d*_ (see Fig. 8). Naturally, this structure is very fragile for the removal of hubs. With increasing robustness 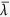, the index *E* of the optimal networks decreases monotonously, and the degree assortativity of network is enhanced. As shown in Fig. 7, links between high-degree nodes and low-degree nodes obviously decreases, and links among high-degree nodes and links among low-degree nodes increases. The multi-hubs star-like structure is gradually changed into a core-periphery structure. In this structure, the interconnected high-degree nodes form the core, while the periphery is composed of interconnected low-degree nodes. Increasing 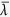, reduce the number of links between the core and the periphery. The core-periphery structure gradually evolves into a core-chain structure. The link density of the core increases, and the periphery expands, and then evolves into long chain or ring substructures (see Fig. 7(c)). The increasing variation of clustering coefficient *C*, network diameter *D* and standard deviation of distance distribution *σ*_*d*_ validates the transition process of network topology. In addition, we find that as the increase of robustness, the spectral gap *SG* increases. It indicates that the optimization for robustness will improve the communicability and the expansion of network.

The results also show that the network with both high robustness and small-world effect exhibits a core-periphery structure, which is quite similar to the results by Netotea and Pongor^39^. It is obviously a compromised structure between the multi-hubs star-like structure and the core-chain structure. Optimization for robustness will make the core denser, and expand the chains of the periphery, while optimization for small-world effect make the core sparser, and fragment the periphery so that each low-degree node connects to one or more hubs.

## Discussion

In this paper, we have emphasized that both robustness and small-world effect are of great importance for designing or optimizing the network topology. Generally, small-world effect can be interpreted by the reciprocal of average shortest path lengths, while robustness is positively related to the redundancy of alternative routes in network which can be precisely measured by natural connectivity.

We have verified that robustness and small-world effect are in a conflicting relation in optimization while conserving node degree by means of large amount of optimization experiments. The efficient network shows a multi-hubs star-like structure, which is proved to be fragile for the removals of high-degree nodes. Conversely, the robust network has a core-chain topology in which high-degree with a cluster of high-degree nodes comprise the core and other low-degree nodes that are interconnected to form long chain substructures. Obviously, such long chain substructure has a serious problem with the communication among nodes.

To optimize both robustness and small-world effect, we proposed a multi-objective evolutionary algorithm including crossover, mutation and reduce operators. We have demonstrated that our algorithm can optimize the network topology for both robustness and small-world effect. We found the networks that realize the trade-off between robustness and small-world effect exhibit a core-periphery structure where high-degree nodes comprise the core and low-degree nodes form the periphery. Optimizing robustness will strengthen the link density of core and expand the periphery, while optimization for small-world effect will weaken the core and fragment the periphery. We also investigate several primary topological properties in optimized networks for robustness and small-world effect to discuss their relation.

As a final point, we note that constraints such as geography or rewiring limitations should be considered in practical applications. When such constraints are imposed, the multi-objective optimization results could potentially be quite different. Moreover, the trade-off between robustness and small-world effect in directed or weighted network are also interesting future directions.

## Methods

### Robustness, small-world effect and topology metrics of network

In this paper, we employ a rather new robustness measure—natural connectivity 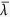
—which has been received growing attention^41^,^42^,^49^,^50^,^51^,^52^,^53^,^54^. Natural connectivity, derived from the Estrada index^42^,^52^, has an intuitive physical meaning and a simple mathematical formulation. Physically, it characterizes the redundancy of alternative paths by quantifying the weighted number of closed walks of all lengths and can also be interpreted as the Helmholtz free energy of a network^41^,^42^. Mathematically, the natural connectivity can be derived from the graph spectrum as an average eigenvalue and increases strictly monotonically with the addition of edges. It is defined as:


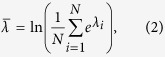


where *N* is the number of nodes in a network *G* and *λ*_*i*_ is the *i*th element of the set {*λ*_1_, *λ*_2_, …, *λ*_*N*_}, which is called the spectrum of *G*. It is shown that the natural connectivity has a strong discrimination in measuring the robustness of complex networks and lower computation complexity. The natural connectivity 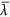 of a network increases in the optimization process, the robustness of the network is improved. Similarly, the network become more fragile as the natural connectivity 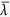 decreases.

To measure the extent of small-world effect, as an alternative to the average path length *L* of a network, we employ the reciprocal harmonic average of shortest distances, also known as efficiency^43^,


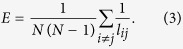


where *l*_*ij*_ is the geodesic distance from node *v*_*i*_ to node *v*_*j*_. We do not use the average path length *L* or other index related to distances considering all-walks (e.g. Estrada community index^52^), because the reciprocal harmonic average of shortest distances has a simple definition and desirable mathematical properties. For example, it is normalized to a range of [0,1] and is valid for disconnected networks. We remark that it can be related to the Harary index *H(G*)^55^, which is a measure of the compactness of the molecule, as follows


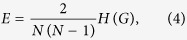


where 
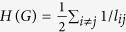
. The index *E* is a summary statistic that combines aspects of the sizes of connected components and the average distances within these. Although it is a quite an arbitrary combination of these more fundamental properties (component size and distance), many authors have argued that it is a powerful predictor for how well the both natural and man-made networks function. It is clear, the small-world effect decreases with both increasing fragmentation (especially splitting large components into similar sizes) and increasing distances. The increase of index *E* indicates that the information exchanges more fast among nodes in the network. Conversely, the decrease of *E* means the loss of small-world effect.

To describe the structure of our optimized networks, we outline a list of standard network topology metrics: the assortativity coefficient *r*, clustering coefficient *C*, network diameter *D*, standard deviation of distance distribution *σ*_*d*_ and spectral gap *SG*. The assortativity coefficient *r* is a measure of assortative mixing by degree in networks, while the clustering coefficient of a node is the fraction of connected node pairs in the node’s neighborhood. The network diameter *D* (the maximal distance among node pairs), average shortest path length *L* and standard deviation of distance distribution *σ*_*d*_ are utilized to measure the characteristics of distance distribution. Spectral gap (*SG*), the difference between the two largest eigenvalues of the adjacency matrix of a network, is also an important metric for network features. For instance, the good expansion (GE) properties in complex networks is shown to be related to the network spectral gap^56^.

### Multi-objective evolutionary optimization algorithm

To solve the multi-objectives optimization model, we utilize an applicable optimization algorithm, S-metric selection evolutionary multi-objective optimization algorithm (SMS-EMOA)^57^. SMS-MOEA is proved to be suited for optimization with two and three objectives. It is better than the conventional algorithms such as SPEA2 in convergence and distribution uniformity. Here we describe the three primary parts of SMS-EMOA for network robustness and small-world effect: crossover operator, mutation operator and reduce operator.

#### Crossover operator

**Figure 9:**
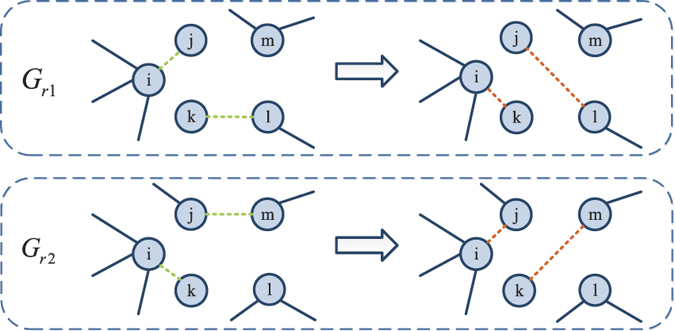
The process of crossover operator. Firstly, two networks *G*_*r*1_ and *G*_*r*2_ are randomly selected for crossover operator. Suppose *V*_*i*_(*G*_*r*1_) is the set of neighbors of node *i* in *G*_*r*1_. And 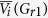 is the set of nodes which connect to node *i* in *G*_*r*1_ but disconnect to node *i* in *G*_*r*2_. Similarly, *V*_*i*_(*G*_*r*2_) and 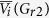 can be obtained. Here 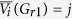 and 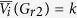. Then randomly select a node *m* in *G*_*r*2_ which connects to node *j* but disconnects to node *k*. In *G*_*r*1_, links *e*_*ij*_ and *e*_*kl*_ are removed, and links *e*_*ik*_ and *e*_*jl*_ are added. In *G*_*r*2_, links *e*_*ik*_ and *e*_*jm*_ are removed, and links *e*_*ij*_ and *e*_*km*_ are added. The green dot lines represent the selected links for cross operator in original network. The red dot lines represent the generated links after cross operator.

The crossover operator fuses the genetic information from a pair of chromosomes and generate a new chromosome at each iteration. When we swap the local structure information from two selected networks, the main constraint we should consider is to keep each node degree fixed after crossover operation. We use an improved version of the crossover operator proposed in ref. 58. Figure 9 shows the process of crossover operator on node *i*. If the two networks are unconnected networks, the crossover operator is invalid, and we start the process again. The process of crossover operator on *G*_*r*1_ and *G*_*r*2_ is to employ the crossover operator on each node in networks according to the crossover probability *p*_*c*_. When the crossover operator is finished, one of the new generated networks is randomly selected as the new solution.

#### Mutation operator

Mutation operator aims to search new solutions in a local area, which can accelerate the convergence of the algorithm. The neighborhood of the current network is the set of networks that are obtained by rewiring the current network while keeping degrees fixed. At each iteration, the rewiring process shown in Fig. 1 is executed as the mutation operation.

#### Reduce operator

When a new network is added to the population, the inferior solution in the population should be removed. Unlike the conventional algorithm, SMS-EMOA does not maximize the objectives individually, but maximizes the hypervolume spanned by them in the space of objectives. The hypervolume is the area under the Pareto-curves and bounded by a reference point^59^. During the reduce operation, the contributing hypervolumes of each solutions is be calculated, and the inferior solution with lowest contributing hypervolume is eliminated from current population. In the optimization model, hypervolume of solution *p*_*i*_ can be calculated by:


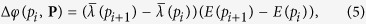


where 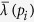 is the robustness of solution *p*_*i*_ and *E(p*_*i*_) is the small-world effect of solution *p*_*i*_. **P** = {*p*_1_, *p*_2_, …, *p*_*v*_} is the current population which is arranged by the order of the value of robustness 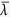 from low to high.

At each iteration, a new solution is generated from the current population by executing the mutation and crossover operations sequentially. Then the solution which has the lowest contribution to the hypervolume is removed. By repeatedly executing the steps above within a large enough number of iterations, a best possible set of non-dominating solutions, called Pareto-optimal front, is obtained.

## Additional Information

**How to cite this article**: Peng, G.-S. *et al.* Trade-offs between robustness and small-world effect in complex networks. *Sci. Rep.* **6**, 37317; doi: 10.1038/srep37317 (2016).

**Publisher’s note**: Springer Nature remains neutral with regard to jurisdictional claims in published maps and institutional affiliations.
